# Genetic epidemiology of motor neuron disease-associated variants in the Scottish population

**DOI:** 10.1016/j.neurobiolaging.2016.12.013

**Published:** 2017-03

**Authors:** Holly A. Black, Danielle J. Leighton, Elaine M. Cleary, Elaine Rose, Laura Stephenson, Shuna Colville, David Ross, Jon Warner, Mary Porteous, George H. Gorrie, Robert Swingler, David Goldstein, Matthew B. Harms, Peter Connick, Suvankar Pal, Timothy J. Aitman, Siddharthan Chandran

**Affiliations:** a Centre for Genomic and Experimental Medicine, Institute of Genetics and Molecular Medicine, University of Edinburgh, Edinburgh, UK; b The Euan MacDonald Centre for Motor Neurone Disease Research, University of Edinburgh, Edinburgh, UK; c Centre for Clinical Brain Sciences, University of Edinburgh, Edinburgh, UK; d South East Scotland Genetics Service, Western General Hospital, Edinburgh, UK; e Institute for Genomic Medicine, Columbia University, New York, USA

**Keywords:** Motor neuron disease, Amyotrophic lateral sclerosis, TBK1, NEK1

## Abstract

Genetic understanding of motor neuron disease (MND) has evolved greatly in the past 10 years, including the recent identification of association between MND and variants in *TBK1* and *NEK1*. Our aim was to determine the frequency of pathogenic variants in known MND genes and to assess whether variants in *TBK1* and *NEK1* contribute to the burden of MND in the Scottish population. *SOD1*, *TARDBP*, *OPTN*, *TBK1*, and *NEK1* were sequenced in 441 cases and 400 controls. In addition to 44 cases known to carry a *C9orf72* hexanucleotide repeat expansion, we identified 31 cases and 2 controls that carried a loss-of-function or pathogenic variant. Loss-of-function variants were found in *TBK1* in 3 cases and no controls and, separately, in *NEK1* in 3 cases and no controls. This study provides an accurate description of the genetic epidemiology of MND in Scotland and provides support for the contribution of both *TBK1* and *NEK1* to MND susceptibility in the Scottish population.

## 1 Introduction

Motor neuron disease (MND) is a rapidly progressive and fatal neurodegenerative disorder, characterized by loss of motor neuron function. Presentations can include limb onset, bulbar onset, or cognitive/behavioural disease, reflecting variable involvement of the upper motor neurons, lower motor neurons and frontotemporal cortex (Turner and Swash, 2015). Age and site of onset, rate of disease progression and clinical syndrome vary considerably between cases, presenting difficulties for diagnosis and disease management.

Phenotype data from Scottish MND cases are available through the Scottish Motor Neuron Disease Register (SMNDR), a prospective population-based record of all cases diagnosed with MND, which has been operational since 1989 (The Scottish Motor Neuron Disease Research Group, 1992). In Scotland, there is a predicted annual crude incidence of MND of 2.38 per 100,000 of the population (Forbes et al., 2007). Mean survival from symptom onset to death is 2.8 years (Forbes et al., 2007), albeit with a variable trajectory depending on the clinical syndrome.

Substantial progress in understanding the genetic landscape of MND has occurred over the last 10 years, including association with dominant variants at several genetic loci (Renton et al., 2014). Although the majority of cases of MND present without a family history (∼90%), variants in the same genes are thought to contribute to the genetic etiology of both familial and apparently sporadic cases. In recent series, pathogenic variants in known genes have been found in around 70% of the cases with a family history and 10% of the cases with no family history (Renton et al., 2014). The main contributors in the UK and other European populations are expansions of an intronic hexanucleotide repeat in *C9orf72* and missense variants in *SOD1* and *TARDBP* (Abel, 2016, Abel et al., 2013, Renton et al., 2014). Although the incidence of MND-associated variants specifically in the Scottish population is unknown, screening of an unselected Scottish cohort for variants in *SOD1* identified a high frequency of the I114T variant, which was found in 9% cases (Jones et al., 1995), and more recently *C9orf72* expansions were found in 11% of Scottish cases (Cleary et al., 2016). Two genes, *TBK1* and *NEK1*, have recently been associated with MND, with each thought to account for a small proportion of cases, although they have not been screened in the Scottish MND population (Brenner et al., 2016, Cirulli et al., 2015, Freischmidt et al., 2015, Kenna et al., 2016).

In families where affected individuals carry the same MND-associated variant, for example *SOD1* I114T, the phenotype can be extremely variable (Lopate et al., 2010). This phenotypic heterogeneity, along with previous reports of pathogenic variants in more than one MND gene in the same affected individual (oligogenic cases; Bury et al., 2016, Cady et al., 2015, Chio et al., 2012b, Kenna et al., 2013, Lattante et al., 2012, van Blitterswijk et al., 2012), has led to a hypothesis that disease risk and subsequent phenotype is determined by a combination of genetic factors and modifiers, rather than a single genetic variant, in combination with environmental triggers, as described in the multistep hypothesis of MND (Al-Chalabi et al., 2014).

In this study, we report a multi-gene screen of MND cases and controls from the Scottish population. Our aim was to determine the contribution of variants in different genes to cases of MND in Scotland, to investigate whether variants in *TBK1* and *NEK1* contribute to the burden of cases and to assess the association of variants in MND genes with disease phenotype.

## 2 Materials and methods

### 2.1 MND case recruitment and control samples

MND cases were recruited through the SMNDR. The registration process achieved high ascertainment coverage (98% in 1989–98; Forbes et al., 2007), providing a cohort representative of the national MND population. Details of methodology of recruitment have been reported previously (The Scottish Motor Neuron Disease Research Group, 1992); latterly the El Escorial classification system was adopted (Brooks, 1994, Brooks et al., 2000). Recruited cases included individuals aged ≥16 with probable or definite amyotrophic lateral sclerosis and individuals with MND subtypes (progressive bulbar palsy, progressive muscular atrophy and primary lateral sclerosis). Individuals provided written consent for DNA extraction and genetic studies.

Four hundred forty-one samples, obtained from cases diagnosed with MND in Scotland in the years 1989–2014, were included in this study, which included 3 pairs of related individuals (2 brothers, 2 first cousins, and 2 first cousins once removed). Case records were examined for 7 phenotypic characteristics: sex, age at onset, age at diagnosis, time to diagnosis, duration of disease (until death or final data review [20th April 2016]), site of onset (bulbar or spinal), and family history of MND. Individuals were classified as having a family history of MND if a first, second, or third degree relative had been known to have MND, as for cases. The study did not include the presence of frontotemporal dementia (FTD) alone as a criterion for positive family history. Five individuals were lost to follow-up due to relocation from Scotland, and survival dates were censored to date of last contact. The cohort was screened for expansions of the *C9orf72* intronic hexanucleotide repeat, as described by Cleary et al. (2016). A subset of the cohort had been screened for variants in *SOD1* in several previous studies (Hayward et al., 1998, Jones et al., 1995, Swingler et al., 1995).

Five MND cases, each with a variant in 1 of the 5 genes sequenced, were included as positive controls; these were taken from the cohort described by Cirulli et al. (2015). Four hundred ethnicity and sex-matched healthy controls were selected from the Generation Scotland Donor DNA databank (Kerr et al., 2010). The selected controls were aged ≥56 at the time of collection (20% aged 50–56, 66% aged 60–65, 14% aged 66+); an older cohort was chosen to minimize inclusion of young subjects who could go onto develop MND later in life.

### 2.2 Targeted amplification and sequencing

Five genes were sequenced: *SOD1*, *TARDBP*, *OPTN*, *TBK1*, and *NEK1*; these will be referred to hereafter as the MND gene panel. The panel includes the recently associated *TBK1* and *NEK1*, alongside genes that are among the largest contributors to cases in UK and European populations (*SOD1*, *TARDBP*, and *OPTN*), after *C9orf72* (Abel, 2016, Abel et al., 2013, Renton et al., 2014).

Primers for 120 amplicons (Supplementary Table 1) were designed according to the Fluidigm Access Array protocol (Fluidigm). The primers amplified the coding regions of these 5 genes (Supplementary Table 2), excluding a total of 312bp of coding sequence across the 5 genes, due to primer design constraints. The Fluidigm Access Array was used for amplification, following the manufacturer's multiplex amplicon tagging protocol, with 2 modifications (Martyna Adamowicz-Brice, personal communication); an additional 1:1 AMPure XP (Agencourt) cleanup step was introduced post-harvesting and the subsequent dilution step was removed. The amplicon library was sequenced on an Illumina MiSeq with 2×150-bp reads. Each batch of 48 samples included at least one water blank as a negative control. The positive control samples were each run in 2 independent batches.

### 2.3 Read mapping, sample filtering, variant calling, and annotation

Quality control of the amplicon sequencing data was performed using FastQC (version 0.11.2) (Andrews, 2010). Primer sequences were removed from the 5′ ends of reads using the cutadapt tool (version 1.7.1) with the ‘anchor’ option (Martin, 2011). The maximum error rate for primer sequences was set to 10%. Reads were mapped to the human genome reference sequence hs37d5 using BWA MEM (version 0.7.10; Li, 2013). Picard (version 1.85; Broad Institute), and DepthOfCoverage GATK tool (version 3.3–0; McKenna et al., 2010) were used to collect alignment and amplicon coverage statistics. The median amplicon coverage of the negative control sample was used to determine the threshold for including samples from the same batch in further analysis. Only samples with a median coverage that was >10× that of the negative control were included. This removed 7 cases and 11 controls, leaving 434 cases (432 independent) and 389 controls for further analysis. The UnifiedGenotyper tool, as implemented in GATK version 2.6 (McKenna et al., 2010), was used for variant calling. ANNOVAR (version 2014 Nov 12) was used to provide functional annotation of the variants (Wang et al., 2010). Variants were annotated with reference to the transcripts listed in Supplementary Table 2.

### 2.4 Variant filtering and validation

Intronic and synonymous variants, variants with a population frequency greater than 1% in either the 1000 Genomes (October 2014 release; 1000 Genomes Project Consortium et al., 2015) or ExAC v0.2 (Lek et al., 2016) data sets and variants with a frequency >5% in our cohort were excluded from further analysis. Per-sample variant calls were filtered to exclude calls with a read depth <50 or an allele balance <0.3. Filtered variants were validated by Sanger sequencing. One sample failed to amplify using PCR at variant validation stage and was excluded from further analysis, leaving 433 (431 independent) cases. Nine false positives were identified in *SOD1* exon 1, and this exon was excluded from the analysis. Following exclusion of this exon, 5 false positives remained, which were excluded from downstream analysis. Validated variants were submitted to Clinvar. The known variants in the positive controls were identified in both assays in which they were tested.

### 2.5 Assessing variant pathogenicity

Stop-gain, frameshift, and splice site variants were categorized according to their predicted effect on protein function. Missense variants reported as disease-causing in association with MND in the Human Gene Mutation Database (Stenson et al., 2008) were categorized as pathogenic. The remaining missense variants were categorized according to *in silico* scores of pathogenicity and conservation. The scores used are listed below, with the thresholds for supporting pathogenicity/conservation in brackets: SIFT (=D), PolyPhen HDIV (=P/D), LRT (=D), Mutation Taster (=D), Mutation Assessor (=M/H), FATHMM (=D), CADD phred (>15), GERP (>2), phyloP (>2), and SiPhy (>10). Variants for which 7–10 *in silico* measures supported pathogenicity/conservation were categorized as likely pathogenic, variants with 4–6 measures supporting pathogenicity/conservation were categorized as uncertain significance, and variants with 0–3 measures supporting pathogenicity/conservation were categorized as likely benign (Supplementary Table 3).

### 2.6 ExAC reference exomes

The ExAC v0.3.1 reference exome data (Exome Aggregation Consortium (ExAC), Lek et al., 2016) were used to provide an additional, larger control population. It contains exome-wide variants identified in 60,706 individuals. For both *TBK1* and *NEK1*, the total number of loss-of-function or loss-of-function + missense alleles in ExAC were recorded (including filtered [non-pass] variants), excluding variants found in >1% individuals. The number of ExAC individuals was reduced to reflect the average percentage of individuals covered at 30× across each gene. For *TBK1*, an average of ∼69% of individuals was covered at 30×, giving 41,848 individuals assumed to have sufficient depth for variant calling. For *NEK1*, it was ∼50%, giving 30,298 individuals.

### 2.7 Statistical analysis

Fisher's exact tests were used in comparisons between the number of variants in cases and controls (Sections 3.2, 3.4 and 3.5). One-tailed tests were used because of the prior assumption that cases contain more pathogenic variants than controls. Genotype-phenotype association testing was used to compare clinical phenotypes with different MND genotypes (Section 3.7). Only unrelated individuals (n = 431) were included in the analysis, with the first individual recruited to the SMNDR from each pair retained for analysis. Variables were examined for collinearity using Pearson's correlation coefficient. Age of onset and age at diagnosis were highly correlated (Pearson's correlation r = 0.98, *p* < 5 × 10^−7^), as were duration of disease from onset and duration of disease from diagnosis (r = 0.87, *p* < 5 × 10^−7^). Disease onset is more important to disease biology than date of diagnosis, which relies on clinical services; therefore, variables related to diagnosis were excluded from genotype-phenotype association testing. Time to diagnosis was also excluded from genotype-phenotype analysis, as this is a derived variable. Univariate analysis was carried out for the following variables: sex, age of onset, duration of disease from onset, site of onset, and family history. Two-tailed Fisher's exact test was used for categorical data and *t*-test or Mann Whitney *U* test was used for parametric and non-parametric continuous data, respectively (Section 3.7). Variables with significant univariate association at *p* ≤ 0.1 were inputted into binomial logistic regression models dependent on gene. Logistic regression analysis was used to test the hypothesis that significant variables were independently associated with having a pathogenic or loss-of-function variant in one of the genes tested. For the purposes of the model, age of onset was grouped by decade. Results from logistic regression modeling were considered significant if *p* < 0.05 (Section 3.7). SPSS Statistics version 21 was used for all statistical analysis.

## 3 Results

### 3.1 MND case phenotypes

After sample filtering, 433 MND cases (431 independent) remained, of which complete phenotypic data were obtained for 428 (99%; Table 1). Site of onset was unrecorded for 1 case. Family history was unrecorded for 3 cases and was unknown for 1 case, who was adopted. The cohort had a male to female ratio of 1.4:1 (Table 1). The mean age of onset was 59.5 years. Of the 443 cases in this cohort, 367 (85%) had died before censorship date; median duration of disease from onset for all the cases was 42 months. Of the 430 independent cases for which site of onset was recorded, 304 (71%) had spinal onset MND. Of the 429 cases with recorded family history, 44 (10%) had a family history of MND.

### 3.2 Identification of variants in MND genes

Targeted sequencing with the MND gene panel achieved a mean coverage of 6280× per amplicon per sample. Following variant filtering and validation (Section 2.4), at least one rare stop-gain, splice site, frameshift, or missense variant was identified in 57/433 cases and 19/389 controls (Supplementary Tables 4 and 5). Following sample filtering (Section 2.3), the case population contained 2 pairs of related individuals. The first pair (brothers) both carried the *SOD1* G94R variant, whereas the second pair (first cousins) did not carry any variants in the MND gene panel that passed variant filtering. Therefore, the results for the related individuals were concordant.

Thirty-seven unique variants were identified in either cases or controls across the MND gene panel; of these, 3 were stop-gain, 3 were splice site, 3 were frameshift, and 28 were missense variants (Supplementary Table 4). All stop-gain, splice site, and frameshift variants were categorized as loss-of-function, except for 3 variants. A stop-gain variant in *TARDBP* was categorized as uncertain significance, as it is in the final exon of the gene and therefore not expected to result in nonsense-mediated decay. There were also 2 splice site variants in *NEK1* predicted to result in the in-frame loss of a single exon (Brunak et al., 1991), which were categorized as uncertain significance. Therefore, of the 37 unique variants identified, 6 were categorized as loss-of-function, 7 pathogenic, 11 likely pathogenic, 8 uncertain significance, and 5 likely benign (Supplementary Table 4).

**Fig. 1:**
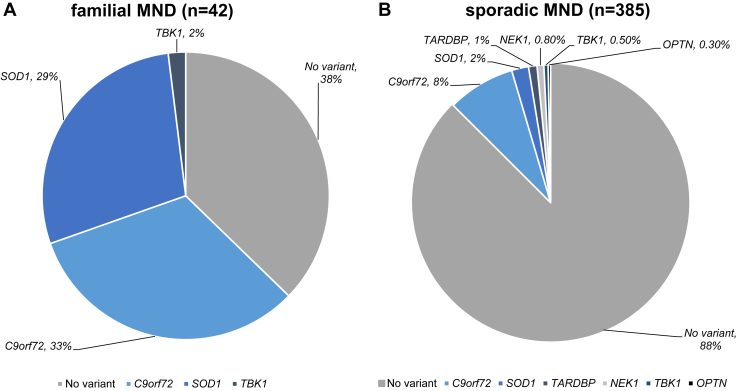
Proportion of cases with a pathogenic or loss-of-function variant in genes on the MND gene panel or in *C9orf72*. (A): cases with a family history; (B): sporadic cases with no known family history. Each pie chart includes one digenic case that is counted for both of the genes in which a pathogenic or loss-of-function variant is found.

In total, 31 MND cases and 2 controls carried at least one loss-of-function or pathogenic variant across the MND gene panel (Table 2 and Supplementary Table 5; Fisher's *p* = 1.761 × 10^−7^). Taken together with prior findings that 44 of the 431 independent cases contain a pathogenic intronic hexanucleotide repeat expansion in *C9orf72* (≥100 repeats; Cleary et al., 2016), 74 independent cases (17%) carried at least one pathogenic or loss-of-function variant (Table 2 and Supplementary Table 5). This represented 26/42 (62%) of cases with a family history and 47/385 (12%) of cases with no family history (Fig. 1).

### 3.3 Variants in *SOD1*, *OPTN*, and *TARDBP*

After *C9orf72*, the largest genetic contributor to cases in our study was *SOD1*, with pathogenic or likely pathogenic variants observed in 22/431 independent MND cases (5%) and 1/389 controls (0.3%; Table 2 and Supplementary Tables 4 and 5). The I114T Scottish founder mutation was observed in 18 cases (4%) and 1 control (0.3%). Of all cases, 29% of familial and 2% of sporadic carry a pathogenic variant in *SOD1*.

Pathogenic variants in *TARDBP* were observed in 4/431 (1%) cases and no controls. This is in addition to a stop-gain variant of uncertain significance, observed in one case. For *OPTN*, at least one pathogenic or likely pathogenic variant was observed in 3/431 (1%) cases and 1/389 controls (0.3%). The single pathogenic variant in *OPTN*, Q314L, was observed in 1 case and 1 control. There is no functional evidence to support the association of this variant with MND and, although in previous studies *in silico* evidence has suggested a pathogenic role for the variant in MND (Del Bo et al., 2011), the occurrence of the variant in 1 case and 1 control in our data suggests that the variant either has variable penetrance or is not pathogenic for MND. *OPTN* therefore appears to contribute to very few cases of MND in the Scottish population.

### 3.4 Assessing the association of variants in *TBK1* with MND

Several previous studies have reported dominant variants in *TBK1* in cases of MND, FTD, and MND-FTD, accounting for approximately 1% of all cases (Borghero et al., 2016, Cirulli et al., 2015, Exome Aggregation Consortium (ExAC), Freischmidt et al., 2015, Gijselinck et al., 2015, Le Ber et al., 2015, Pottier et al., 2015, Shu et al., 2016, Tsai et al., 2016, van Rheenen et al., 2016, Williams et al., 2015). In our study, we observed 6 cases and 1 control with a rare stop-gain, splice site frameshift, or missense variant in *TBK1* (Section 2.4; Table 2 and Supplementary Table 5; Fisher's *p* = 0.080). When compared with the ExAC reference data set (Exome Aggregation Consortium (ExAC), Lek et al., 2016), this result is not significant (Fisher's *p* = 0.238). When looking only at loss-of-function variants, 3 cases and no controls contained a loss-of-function variant in *TBK1* (Fisher's *p* = 0.145). When compared with the ExAC reference data set, where the frequency of loss-of-function variants was 0.03%, this represents a statistically significant excess (Fisher's *p* = 4.370 × 10^−4^; Exome Aggregation Consortium (ExAC), Lek et al., 2016).

### 3.5 Assessing the association of variants in *NEK1* with MND

Loss-of-function variants in *NEK1* have been associated with MND in two recent studies of both familial and sporadic MND cases (Brenner et al., 2016, Kenna et al., 2016), following an earlier study of mostly sporadic cases that highlighted *NEK1* as a candidate gene (Cirulli et al., 2015, Exome Aggregation Consortium (ExAC)). These 3 studies estimate that loss-of-function variants in *NEK1* contribute to approximately 1% of cases. In this study, we observe 3 cases and no controls with a variant predicted to result in loss-of-function of *NEK1* (Table 2 and Supplementary Table 5; Fisher's *p* = 0.145). When compared with the ExAC reference data set, where the frequency of loss-of-function variants was 0.3%, our result remains nonsignificant (Fisher's *p* = 0.143; Exome Aggregation Consortium (ExAC), Lek et al., 2016). Regarding missense variants in *NEK1* (Section 2.4; Table 2 and Supplementary Table 5), we observe 16 cases and 16 controls with an *NEK1* missense variant, which is not statistically significant (Fisher's *p* = 0.453).

### 3.6 Cases carrying 2 variants in MND-associated genes

There are several reports of MND cases that carry MND-associated variants in more than 1 gene (Bury et al., 2016, Cady et al., 2015, Chio et al., 2012b, Kenna et al., 2013, Lattante et al., 2012, van Blitterswijk et al., 2012). We observed 5 cases and 1 control carrying 2 different rare stop-gain, frameshift, splice site, or missense variants (Section 2.4) either across the MND gene panel or in *C9orf72* (Table 3). Two cases, MND-0040 and MND-0434, and no controls carry 2 variants that are predicted to be either pathogenic or loss-of-function; these will be referred to as digenic cases (Table 3). Both digenic cases have an age of onset within 2 standard deviations of the mean and a typical disease duration (Table 3). However, as discussed in Section 3.3, the pathogenicity of the variant Q314L in *OPTN* in MND-0040 is unclear.

Of the remaining cases with two rare variants in MND-associated genes, case MND-0119 carries 1 pathogenic variant and 1 variant of uncertain significance and has a typical age of onset and disease duration. Case MND-0158, who carries 1 pathogenic and 1 likely pathogenic variant, had a young age of onset (26 years, >2.5 standard deviations from the mean) and long disease duration (142 months, above 90th percentile). MND-0211 also carries 1 pathogenic and 1 likely pathogenic variant and had a typical age of onset, but disease duration at the lower end of the spectrum (16 months, equal to the 10th percentile). Interestingly, 3 of the 5 cases (60%) carrying 2 rare stop-gain, splice site, frameshift, or missense variants in MND genes (including *C9orf72* expansions) had a bulbar site of onset, which is higher than the 29% observed across all MND cases in the cohort.

CONTROL-0325, who was aged 56–60 at the time of collection, carries 2 missense variants in *OPTN*; one considered pathogenic; and 1 likely pathogenic. The pathogenicity of variant Q314L, as discussed in Section 3.3, is unclear. The data available do not allow confirmation of whether the 2 *OPTN* variants are found in *cis* or in *trans* and, therefore, if both allelic copies of *OPTN* carry a variant. It is also unclear whether both variants are expected to contribute to MND pathogenesis; if so, they would be expected to be associated with variable penetrance. As no follow-up information is available for controls, there is a small chance the control developed MND after sample donation, although this is unlikely given the lifetime risk of MND.

### 3.7 Genotype-phenotype associations

Genotype-phenotype association testing was used to determine the relationship between carrying a variant in a specific gene and 5 phenotypic markers (Table 4 and Supplementary Table 6). MND cases carrying the *SOD1* I114T founder variant were also analyzed independently of other *SOD1* variants in view of the high incidence of this specific variant in the Scottish population. Only unrelated cases were included in the analysis (n = 431).

Univariate analyses comparing cases with a pathogenic or loss-of-function variant (including *C9orf72* expansions) to cases without such a variant found associations between carrying a pathogenic or loss-of-function variant and a family history of MND (Fisher's *p* < 5 × 10^−7^), a younger age of onset (*t*-test *p* = 0.018), and female sex (Fisher's *p* = 0.028; Table 4). A logistic regression model evaluating the possible independent associations of these variables with variant status explained 21% of the variance between cases with and without a pathogenic or loss-of-function variant (Nagelkerke R^2^). Independent associations were found for positive family history (*p* < 5 × 10^−7^, OR 10.88, 95% CI 5.38–22.01), decreasing age of onset by decade (*p* = 0.018, OR 0.77, 95% CI 0.62–0.96), and female sex (*p* = 0.028, OR 1.88, 95% CI 1.07–3.30; Table 5).

A high proportion of cases carrying a pathogenic *SOD1* variant had spinal onset disease (95%) compared with the frequency of spinal onset in all other case (Fisher's *p* = 0.012). Carrying a pathogenic *SOD1* variant was also associated with having a family history of MND, compared to all other cases (Fisher's *p* < 5 × 10^−7^; Table 4). When examining *SOD1* I114T carriers in isolation, these 2 factors remained significant (site of onset Fisher's *p* = 0.031, family history Fisher's *p* = 9 × 10^−6^; Table 4). Twenty-seven percent of the variance between *SOD1* pathogenic variant carriers and all other cases was explained by the model (Nagelkerke R^2^). Spinal onset disease (*p* = 0.044, OR 8.25, 95% CI 1.06–64.49) and family history of MND (*p* < 5 × 10^−7^, OR 16.21, 95% CI 6.21–42.33) were independently associated with carrying a *SOD1* pathogenic variant. After initial modeling for the *SOD1* I114T variant (using sex, site of onset, and family history), sex and site of onset did not achieve significance at the *p* = 0.1 threshold and were removed. On revised modeling, family history remained significant (*p* = 1 × 10^−6^, OR 11.39, 95% CI 4.23–30.67; Table 5), explaining 16% of the variance (Nagelkerke R^2^).

Univariate analysis showed carrying a *C9orf72* expansion was associated with having a family history of MND (Fisher's *p* = 1 × 10^−5^; Table 4). Site of onset did not reach statistical significance (*p* < 0.05), but met the criteria required for inclusion in the logistic regression model (Fisher's *p* = 0.082; Table 4). The logistic regression model explained 12% of the variance between carriers of *C9orf72* expansions and those without (Nagelkerke R^2^). Bulbar onset disease (*p* = 0.021, OR 2.23, 95% CI 1.13–4.42) and family history (*p* = 1 × 10^−6^, OR 6.86, 95% CI 3.19–14.77) were significantly and independently associated with carrying a *C9orf72* expansion (Table 5).

**Fig. 2:**
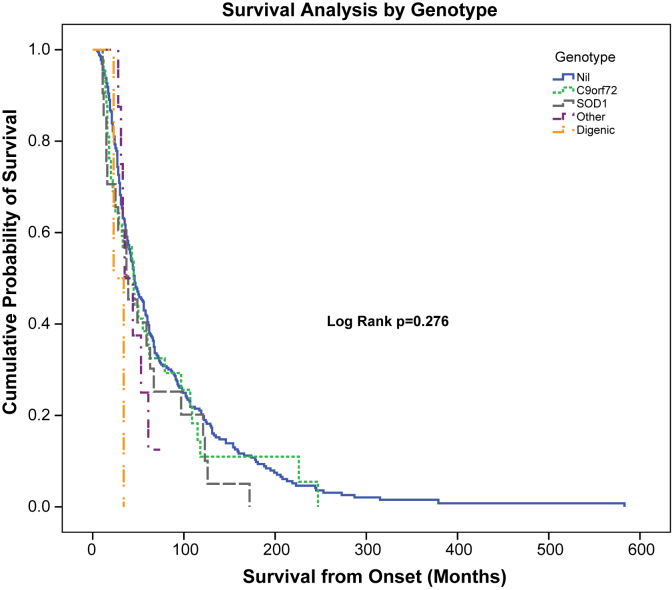
Kaplan-Meier survival plot of MND cases grouped by genotype. *C9orf72*, pathogenic expansion identified; digenic, two pathogenic or loss-of-function variants identified; Nil, no pathogenic or loss-of-function variant identified; other, pathogenic or loss-of-function variant identified in *TARDBP*, *TBK1*, *NEK1*, or *OPTN*; *SOD1*, pathogenic *SOD1* variant identified.

The influence of carrying a pathogenic or loss-of-function variant in one of the MND genes on survival was visualized using a Kaplan-Meier plot (Fig. 2). Cases were grouped by genotype. Survival was plotted for all cases, including the 15% of cases for whom duration of disease was calculated from onset to date of last contact/study truncation. No significant difference was found between groups (log rank *p* = 0.276).

## 4 Discussion

### 4.1 Genetic epidemiology of MND in Scotland

This study provides a detailed description of the genetic pathology of MND in Scotland. We have identified 31 independent cases carrying a pathogenic or loss-of-function variant across our MND gene panel. The identification of pathogenic variants in 2 controls (0.5%) is comparable to previous studies and is explained by the variable penetrance observed with variants in genes associated with MND (Lopate et al., 2010, van Blitterswijk et al., 2012).

The Scottish MND population were comparable with other European MND populations on demographic and disease characteristics (Li et al., 1990, Logroscino et al., 2008, Traynor et al., 2000). A positive family history in 10% of Scottish MND cases was comparable recent population estimates (16% in the Irish population and 9% in a US population of European ancestry; Byrne et al., 2013, Gibson et al., 2014). Our data show that, after *C9orf72*, variants in *SOD1* contribute to the largest proportion of MND cases (5%) in the Scottish population, which is in the upper range compared with other populations of European ancestry (Andersen, 2006, Renton et al., 2014). This reflects the high frequency of the I114T variant, which had previously been identified as a founder mutation with variable penetrance in the Scottish population (Hayward et al., 1996, Jones et al., 1993, Jones et al., 1995, Lopate et al., 2010). Although this variant is less frequent than was observed previously in a smaller cohort (Hayward et al., 1996), it is still found in 4% of cases when assessed across our larger cohort and is found in 24% of those cases in which a pathogenic variant was identified. In comparison, pathogenic variants in *TARDBP* contribute to <1% of cases, comparable to previous estimates in populations of European ancestry (Renton et al., 2014), and pathogenic variants in *OPTN* are extremely rare, which is similar to a previous study of the UK population (Johnson et al., 2012).

Our data support a growing literature associating loss-of-function variants in *TBK1* with MND. Several previous studies have associated dominant coding variants in *TBK1* with MND. The first study reported a significant excess of coding variants in *TBK1*, excluding those predicted to be benign, in mostly sporadic cases compared to controls (Cirulli et al., 2015). However, the biggest excess was observed for loss-of-function variants. This was supported by a second study, which found a significant excess of loss-of-function variants in *TBK1*, but only in familial cases (Freischmidt et al., 2015). Several other studies, looking at MND, frontotemporal dementia (FTD) and MND-FTD cases from both European and Asian populations, have also reported variants in *TBK1,* mostly loss-of-function (Borghero et al., 2016, Gijselinck et al., 2015, Le Ber et al., 2015, Pottier et al., 2015, Shu et al., 2016, Tsai et al., 2016, van Rheenen et al., 2016, Williams et al., 2015). In our study, although the number of loss-of-function variants identified in *TBK1* was not statistically significant when compared with our 389 controls, a significant difference was observed when compared with the larger ExAC reference data set and the percentage of cases carrying a loss-of-function variant in *TBK1* was comparable to previous studies (0.7% vs. <1% in previous studies [Cirulli et al., 2015, Freischmidt et al., 2015]). Therefore, our data support the association of loss-of-function variants in *TBK1* with MND.

Similarly, the frequency of *NEK1* loss-of-function variants in our cases (0.7%) is comparable to previous studies (1%; Brenner et al., 2016, Cirulli et al., 2015, Kenna et al., 2016); however, the observation of 3 loss-of-function variants in *NEK1* in our cases was not significantly different to that of our 389 controls or the ExAC reference data set. Although the result does not provide formal statistical significance for the association of loss-of-function variants in *NEK1* with MND, as the frequency is comparable to other studies, our result is supportive of these previous findings. We observed the same number of missense variants in *NEK1* in both cases and controls, with little difference in the assigned pathogenicity classifications. This is comparable to a recent study which showed that, although one specific missense variant, R261H (classified as likely pathogenic in our study), was associated with MND, collectively, missense variants showed very little difference in frequency between sporadic cases and controls (Kenna et al., 2016). In addition, the other variants in *NEK1* causally related to the development of MND in previous studies are loss-of-function variants. There may therefore be some specific missense variants that result in impaired *NEK1* function, but collectively, this appears not to be the case.

We identified 2 cases each carrying 2 variants classified as pathogenic or loss-of-function in different MND genes (digenic cases). As controls were not screened for hexanucleotide repeat expansions in *C9orf72*, we were biased against identifying digenic controls, so it is not clear whether our 2 digenic cases represent a statistically significant excess. As each digenic case has a different variant combination, it is not possible to obtain a detailed assessment of how digenicity influences MND phenotype. Further investigation of a larger number of digenic cases is required to determine whether each variant contributes to disease pathogenesis, whether variants have an additive effect and to determine how different variant combinations influence disease presentation.

### 4.2 Genotype-phenotype associations

Our genotype-phenotype analysis confirms the expected association that MND cases with a family history of MND are more likely to carry a pathogenic or loss-of-function variant than cases with no family history. The results also indicate that a young age of onset of MND is a significant independent predictor of carrying a pathogenic variant. Although this finding requires replication, it suggests that, in addition to the presence of a family history, clinicians should use a lower threshold for genetic testing in cases developing symptoms of MND before the mean age of onset. The analysis also suggests that female sex is significantly associated with carrying a pathogenic or loss-of-function variant. This most likely reflects the equal number of males and females carrying variants pathogenic for MND, within an MND case population which overall has a male bias. This result is also consistent with the liability threshold genetic model, which would suggest that, as females have an overall lower risk than males of developing MND, they must accumulate a larger burden of risk factors to pass the threshold for disease onset (Falconer, 1965). The logistic regression model concerned only explains 21% of the difference between cases with a pathogenic or loss-of-function variant and those without, suggesting that additional phenotypic markers are acting as confounders.

Our study confirms that *C9orf72* expansion carriers are more likely to present with bulbar disease than other MND cases (Byrne et al., 2012, Chio et al., 2012a, Millecamps et al., 2012). However, our model explained only 12% of the variance, and substantial phenotypic heterogeneity among *C9orf72* carriers, particularly in terms of cognitive symptoms, is widely recognized (Rohrer et al., 2015). Cognitive profile may be an important parameter to explore further in characterizing this cohort of cases. *SOD1* variant carriers in our cohort were relatively homogeneous in terms of site of onset, with a bias toward spinal onset MND. This mirrors clinical accounts of lower limb onset disease as a common presentation of *SOD1* MND (Millecamps et al., 2010). A high proportion of I114T carriers also had spinal onset disease, despite failing to achieve statistical significance on logistic regression modeling. However, heterogeneity in I114T carriers for age of onset (range 42–84 years) and duration of disease from onset (11–172 months) was notable and suggests any biological link to these features is absent or extremely weak.

### 4.3 Limitations

There is increasing need for standardization of methods to assign variant pathogenicity. The recent American College of Medical Genetics guidelines (Richards et al., 2015) sought to address this; however, they are designed principally for monogenic, Mendelian disorders and may not be suitable for complex disorders such as MND, with a preponderance of sporadic cases and considerable variability in the penetrance of pathogenic variants. The lack of segregation data and large number of singleton variants meant that using *in silico* predictors of variant pathogenicity and conservation were most appropriate for assessing variant pathogenicity in our study, although it is clear that standardization of the methods used to assign pathogenicity for potential MND-associated variants is required.

One limitation of the combined use of the Fluidigm Access Array and Illumina MiSeq is the number of false positives we observed. The large number in *SOD1* exon 1 is most likely due to the high GC content of this exon (Timothy J Aitman, unpublished data). Exclusion of this exon could have reduced the overall variant detection rate for *SOD1*, as many pathogenic missense variants have been reported in this exon (Stenson et al., 2008). After excluding this exon, 6% of the remaining variants also failed to validate. This could be due to the high number of cycles in the 2 rounds of PCR (35 and 15 cycles respectively) required to generate the sequencing library. Therefore, it is essential to use Sanger sequencing to validate variants identified through this protocol, as is typically applied following high-throughput sequencing.

Survival analysis between MND cases carrying pathogenic or loss-of-function variants in different genes did not achieve statistical significance. Possible limitations to this analysis include sample size and lack of data relating to interventions that influence survival (e.g., noninvasive ventilation, gastrostomy insertion, and use of riluzole).

## 5 Conclusions

In summary, we identified a pathogenic or loss-of-function variant in an MND gene in 17% of our cohort of MND cases from the Scottish population. Our data give supporting evidence for the association of loss-of-function variants in *TBK1* and *NEK1* with MND. Genotype-phenotype association testing has highlighted that MND cases with a family history or with a young age of onset are significantly more likely to carry a genetic variant pathogenic for MND and suggests that cases presenting with a young age of onset should be referred for genetic testing, in addition to cases with a family history.

## Disclosure statement

Timothy J. Aitman is in receipt of speaker honoraria from Illumina and consultancy fees from AstraZeneca. The other authors have no conflict of interests to declare.

## Figures and Tables

**Table 1:** Phenotypic characteristics of MND cases (n = 433)

Phenotypic characteristic	Summary statistic	Values
Sex	Female (%)	177/433 (41)
Age of onset (y)	Mean (SD) Range	59.5 (12.9) 14–94
Age of diagnosis (y)	Mean (SD) Range	61.1 (12.6) 19–94
Time to diagnosis (mo)	Median (IQR) Range	12 (6, 22) 0–386
Duration of disease from onset (mo)	Median (IQR) Range	42 (25, 73.5) 4–583
Duration of disease from diagnosis (mo)	Median (IQR) Range	25 (12, 55.5) 0–309
Site of onset	Bulbar (%)	126/432 (29)
Family history of MND	Yes (%)	44/429 (10)

Duration of disease: survival until (i) death (n = 367), (ii) data censorship at 20th April 2016 (n = 61), or (iii) date of last contact (n = 5). Includes 2 pairs of related individuals.

Key: SD, standard deviation.

**Table 2:** Variants identified across the MND gene panel, with their pathogenicity classification and frequency in cases and controls

Gene	Loss-of-function		Pathogenic		Likely pathogenic		Uncertain significance		Likely benign		Total^b^	
	Cases	Controls	Cases	Controls	Cases	Controls	Cases	Controls	Cases	Controls	Cases	Controls
*SOD1*	0	0	22^a^	1	1	0	0	0	0	0	23^a^	1
*TARDBP*	0	0	4	0	0	0	1	0	0	0	5	0
*OPTN*	0	0	1	1	2	1	1	0	0	0	4	2
*TBK1*	3	0	0	0	2	0	1	1	0	0	6	1
*NEK1*	3	0	0	0	10	8	6	4	2	4	21	16

a Includes 1 pair of brothers.

b The total includes 1 case with a pathogenic variant in *OPTN* and a loss-of-function variant in *TBK1*; a case with a pathogenic variant in *TARDBP* and a variant of uncertain significance in *NEK1*; and a control with a pathogenic and likely pathogenic variant in *OPTN*.

**Table 3:** Samples containing 2 variants that potentially contribute to MND pathogenicity

Sample information		Phenotype information				Variant 1				Variant 2			
Sample ID	Sample type	Age of onset (y)	Duration of disease from onset (mo)	Site of onset	Family history	Gene	Variant	Variant type	Pathogenicity	Gene	Variant	Variant type	Pathogenicity
MND-0040	Case	46	23	Bulbar	No	*OPTN*	c.A941T; p.Q314L	missense	Pathogenic	*TBK1*	c.2114_2126del; p.A705fs	frameshift	Loss-of-function
MND-0434	Case	44	34	Limb	Yes	*C9orf72*	(GGGGCC)n	intronic HRE	Pathogenic	*TBK1*	c.1427delA; p.E476fs	frameshift	Loss-of-function
MND-0158	Case	26	142	Limb	No	*C9orf72*	(GGGGCC)n	intronic HRE	Pathogenic	*TBK1*	c.C829G; p.L277V	missense	Likely Pathogenic
MND-0211	Case	65	16	Bulbar	No	*C9orf72*	(GGGGCC)n	intronic HRE	Pathogenic	*NEK1*	c.T2235G; p.N745K	missense	Likely Pathogenic
MND-0119	Case	64	44	Bulbar	No	*TARDBP*	c.G859A; p.G287S	missense	Pathogenic	*NEK1*	c. G1021A; p.A341T	missense	Uncertain significance
CONTROL-0325	Control	N/A	N/A	N/A	N/A	*OPTN*	c.A941T; p.Q314L	missense	Pathogenic	*OPTN*	c.A280C; p.K94Q	missense	Likely pathogenic

The table includes samples that carry 2 rare stop-gain, splice site, frameshift or missense variants in genes on the MND panel or samples that carry an expansion in *C9orf72* alongside a variant in a gene on the MND gene panel. N/A, not applicable.

**Table 4:** Genotype-phenotype tests of association comparing phenotype with pathogenic or loss-of-function variants in different MND genes

Phenotypic characteristic	Statistic	No Path (n = 357)	Path/LoF (n = 74)	C9orf72 (n = 44)	SOD1 inc. I114T (n = 21)	SOD1 I114T (n = 18)	TARDBP (n = 4)	TBK1 (n = 3)	NEK1 (n = 3)	Digenic (n = 2)
Sex	Female (%) Fisher's	138 (39) -	39 (53) **p = 0.028**	21 (48) p = 0.419	12 (57) p = 0.171	11 (61) **p = 0.090**	3 (75) p = 0.310	2 (67) p = 0.571	1 (33) p = 1.000	1 (50) p = 1.000
Age of onset (y)	Mean (SD) Mean diff CI t-test	60.2 (12.9) -	56.3 (12.1) −0.39 −0.72 to 0.07 **p = 0.018**	55.6 (11.4) −0.44 −0.84 to 0.04 p = 0.266	55.9 (14.4) −0.38 −0.94 to 0.18 p = 0.186	59.4 (12.0) −0.01 −0.62 to 0.59 p = 0.967	61.3 (7.8) 0.18 −1.09 to 1.44 p = 0.786	48.7 (6.4) −1.09 −2.55 to 0.36 p = 0.141	66.7 (10.6) 0.72 −0.74 to 2.18 p = 0.332	45.0 (1.4) −1.46 −3.24 to 0.32 p = 0.108
Duration of disease from onset (mo)	Median (IQR) Mann-Whitney	42.0 (48) -	37.0 (45) U = 11,784.0 p = 0.144	38.5 (53) U = 7644.0 p = 0.266	37.0 (67) U = 3847.5 p = 0.411	38.0 (89) U = 3483.0 p = 0.652	39.5 (17) U = 834.0 p = 0.936	34.0 (-) U = 558.5 p = 0.698	31.0 (-) U = 610.5 p = 0.884	28.5 (-) U = 271.5 p = 0.370
Site of onset	Bulbar % Fisher's	103 (29) -	23 (31) p = 0.779	18 (41) **p = 0.082**	1 (5) **p = 0.012**	1 (6) **p = 0.031**	2 (50) p = 0.584	2 (67) p = 0.207	0 (0) p = 0.559	1 (50) p = 0.501
Family history of MND	Yes (%) Fisher's	16 (5) -	26 (36) **p < 5 × 10**^−7^	14 (33) **p = 1 × 10**^−5^	12 (57) **p < 5 × 10**^−7^	9 (50) **p = 9 × 10**^−6^	0 (0) p = 1.000	1 (33) p = 0.268	0 (0) p = 1.000	1 (50) p = 0.187

Significant associations (p < 0.10) are highlighted in bold. CI, 95% confidence Interval; LoF, loss-of-function; Mean diff, difference in means between group of interest and all other cases; Path, pathogenic variant, or expansion. Full phenotype data are given in Supplementary Table 6.

**Table 5:** Logistic regression modeling of genotype-phenotype tests of association for (i) cases with a pathogenic or loss-of-function variant across the MND gene panel or *C9orf72* expansion (ii) cases with a *C9orf72* expansion (iii) cases with a *SOD1* pathogenic variant (iv) cases with the *SOD1* I114T variant

Variant type	Predictor	*p*-value	OR (CI)
Path/LoF (n = 74)	Female sex	0.028	1.88 (1.07–3.30)
	Age of onset (decade)	0.018	0.77 (0.62–0.96)
	Family history of MND	<5 × 10^−7^	10.88 (5.38–22.01)
*C9orf72* (n = 44)	Bulbar onset	0.021	2.23 (1.13–4.42)
	Family history of MND	1 × 10^−6^	6.86 (3.19–14.77)
*SOD1* (n = 21)	Spinal onset	0.044	8.25 (1.06–64.49)
	Family history of MND	<5 × 10^−7^	16.21 (6.21–42.33)
*SOD1* I114T (n = 18)	Family history of MND	1 × 10^−6^	11.39 (4.23–30.67)

Key: CI, 95% confidence interval; LoF, loss-of-function; OR, odds ratio; path, pathogenic variant or *C9orf72* expansion.
