# Corrigendum to “Genetic epidemiology of motor neuron disease-associated variants in the Scottish population.” [Neurobiol. Aging 51 (2017) 178.e11–178.e20]

**DOI:** 10.1016/j.neurobiolaging.2017.04.019

**Published:** 2017-08

**Authors:** Holly A. Black, Danielle J. Leighton, Elaine M. Cleary, Elaine Rose, Laura Stephenson, Shuna Colville, David Ross, Jon Warner, Mary Porteous, George H. Gorrie, Robert Swingler, David Goldstein, Matthew B. Harms, Peter Connick, Suvankar Pal, Timothy J. Aitman, Siddharthan Chandran

In the above-mentioned article, the authors would like to revise (changes are in bold) the acknowledgement section as follows:

“The authors thank the patients for consenting to research. In addition, they acknowledge Alona Sosinsky and the Imperial College BRC Genomics facility for bioinformatics support. **They acknowledge Generation Scotland for providing control samples and thank Shona Kerr and Archie Campbell for assistance in provision of Generation Scotland samples.** The authors acknowledge Cat Graham for providing statistical guidance and also thank David Parry and Sophie Marion de Proce for providing helpful comments on the manuscript.

The authors acknowledge the MRC funding awarded to Timothy J. Aitman for translational support, the Euan MacDonald Centre for Motor Neurone Disease Research and MND Scotland. Danielle J. Leighton also receives funding from the Chief Scientist Office, Scotland and the MND Association. Peter Connick is funded by the Wellcome Trust. **Generation Scotland has received core funding from the Chief Scientist Office of the Scottish Government Health Directorates (CZD/16/6 and CZB/4/285) and the Scottish Funding Council, HR03006.**

Ethical approvals were obtained for the SMNDR (MREC/98/0/56 1989-2010, 10/MRE00/78 2011-2015, Scotland A Research Ethics Committee 2015-present), the Scottish MND DNA bank (MREC/98/0/56 1989-2010, 10/MRE00/77 2011-2013, approved by the Scotland A Research Ethics Committee) and the Scottish Regenerative Neurology Tissue Bank (including DNA collection; MREC/98/0/56 1989-2010, 10/MRE00/77 2011 to 2013, 13/ES/0126 2013-2015, 15/ES/0094 2015-present, approved by the Chief Scientist Office Scotland). **Ethical approval for the GS:3D study was obtained from the Tayside Committee on Medical Research Ethics (on behalf of the National Health Service), held by the NHS Lothian NRS BioResource (REC ref: 13/ES/0126).”**

